# Stress modulates intestinal secretory immunoglobulin A

**DOI:** 10.3389/fnint.2013.00086

**Published:** 2013-12-02

**Authors:** Rafael Campos-Rodríguez, Marycarmen Godínez-Victoria, Edgar Abarca-Rojano, Judith Pacheco-Yépez, Humberto Reyna-Garfias, Reyna Elizabeth Barbosa-Cabrera, Maria Elisa Drago-Serrano

**Affiliations:** ^1^Sección de Posgrado e Investigación, Escuela Superior de Medicina, Instituto Politécnico NacionalDistrito Federal, México; ^2^Departamento de Sistemas Biológicos, Unidad Xochimilco, Universidad Autónoma MetropolitanaDistrito Federal, México

**Keywords:** SIgA, pIgR, intestinal mucosa, restraint-stress, glucocorticoids, brain-gut axis

## Abstract

Stress is a response of the central nervous system to environmental stimuli perceived as a threat to homeostasis. The stress response triggers the generation of neurotransmitters and hormones from the hypothalamus pituitary adrenal axis, sympathetic axis and brain gut axis, and in this way modulates the intestinal immune system. The effects of psychological stress on intestinal immunity have been investigated mostly with the restraint/immobilization rodent model, resulting in an up or down modulation of SIgA levels depending on the intensity and time of exposure to stress. SIgA is a protein complex formed by dimeric (dIgA) or polymeric IgA (pIgA) and the secretory component (SC), a peptide derived from the polymeric immunoglobulin receptor (pIgR). The latter receptor is a transmembrane protein expressed on the basolateral side of gut epithelial cells, where it uptakes dIgA or pIgA released by plasma cells in the lamina propria. As a result, the IgA-pIgR complex is formed and transported by vesicles to the apical side of epithelial cells. pIgR is then cleaved to release SIgA into the luminal secretions of gut. Down modulation of SIgA associated with stress can have negative repercussions on intestinal function and integrity. This can take the form of increased adhesion of pathogenic agents to the intestinal epithelium and/or an altered balance of inflammation leading to greater intestinal permeability. Most studies on the molecular and biochemical mechanisms involved in the stress response have focused on systemic immunity. The present review analyzes the impact of stress (mostly by restraint/immobilization, but also with mention of other models) on the generation of SIgA, pIgR and other humoral and cellular components involved in the intestinal immune response. Insights into these mechanisms could lead to better therapies for protecting against pathogenic agents and avoiding epithelial tissue damage by modulating intestinal inflammation.

## INTRODUCTION

Stress is a response of the central nervous system (CNS) to environmental stimuli perceived as a threat to homeostasis. The stress response involves a complex network of mechanisms essential for survival, mediated by neurotransmitters, peptidic hormones and endocrine hormones from the enteric nervous system (ENS), a branch of the autonomic nervous system that among other functions affects the production of interleukins (ILs). These molecules in turn modulate the humoral and cellular components of the intestinal immune system. The ENS contains both vagal and spinal sensory neurons, which play an essential role in the transference of information from the CNS to ENS and vice versa (de Jonge, 2013).

Experimental assays have evidenced that the stress modulates the generation of secretory immunoglobulin A (SIgA; Jarillo-Luna et al., 2007; Martínez-Carrillo et al., 2011) and the expression of pIgR (Reyna-Garfias et al., 2010). Through transcytosis this receptor transports immunoglobin-pIgR complexes (dIgA-pIgR and pIgA-pIgR) across gut epithelial cells. Upon reaching the apical side of these cells, pIgR is cleaved to release SIgA or SC, a pIgR derived peptide into the intestinal lumen (Brandtzaeg, 2009). Along with the gut microflora, both SIgA and pIgR have an essential role in two important intestinal processes. They protect against pathogenic agents that colonize and/or invade the intestinal epithelium, and modulate the gut inflammatory response to maintain homeostasis (Michetti et al., 1992; Uren et al., 2003; Sait et al., 2007; Bruno et al., 2010; Drago-Serrano et al., 2010).

Few studies have explored the effect of stress on SIgA and pIgR, or the capacity of these molecules to maintain homeostasis in the intestine. Most studies involving molecular and biochemical mechanisms of the stress response have focused on systemic rather than intestinal immunity.

The aim of the present review is to explore the impact of stress on the humoral and cellular components of the intestinal immune response, especially IgA, SIgA and pIgR. Although most of the evidence is from studies involving rodent models of restraint/immobilization, other stress-producing protocols affecting SIgA are mentioned, including loud noise, alternating home and metabolic cages, exposure to heat, repeated electric foot shock, and the mixing of newborns from distinct litters (**Table 1**).

**Table 1:** Impact of stress on intestinal immunity.

Animal model	Effect	Reference
Alternating home/metabolic cages (male rats)	↓SIgA; fecal/urine corticosterone excretion unchanged	Eriksson et al. (2004)
Heat stress (rats)	↓SIgA; ↓ IL-2,-4, and -10 in small gut; ↑CD8+ T cells in MLN	Liu et al. (2012)
Repeated electric foot shock (mice)	↓IFN-γ released by gut IEL and α/β TCR+ cells; ↑glucocorticoids	Zhang et al. (2005)
Electric foot shock (EFS) and psychological stress (PS) (rats)	↓SIgA (PS); ↑IgA (EFS) in MLN; ↑corticosterone (EFS)	Yamamoto et al. (2009)
Repeated restraint stress (mice)	↓SIgA; lamina propria IgA+ plasm a cell levels unchanged; ↓gut intraepithelial lymphocytes via adrenal hormones	Jarillo-Luna et al. (2007, 2008)
Restraint stress (mice)	↓T cell and B cells; ↑apoptosis in PP; ↑glucocorticoids	Sudo et al. (2001)
Chronic restraint stress (mice)	↓SIgA+ plasma cells, CD8+T and B cells in PP	Martínez-Carrillo et al. (2011)
Immobilization and acoustic stress (CB1R ko mice)	↓SIgA; ↑ bacterial translocation	Zoppi et al. (2012)
Repeated immobilization (rats with MCAO)	↓SIgA; ↑ bacterial translocation, colon inflammation	Caso et al. (2009)
Acute immobilization stress (rats)	↓SIgA; ↑colon inflammation	Ponferrada et al. (2007)
Repeated restraint stress (rats)	↑SIgA and α- chain mRNA in proximal and distal gut	Reyna-Garfias et al. (2010)
Weaning, cold stress, mix of piglets infected with ETEC	↑SIgA and ETEC fecal shedding	Jones et al. (2001)

*↓ down modulation; ↑ up modulation; EFS, electric foot shock; IgA, immunoglobulin A; IFN-γ, interferon-γ; IL, interleukin; IEL, intraepithelial lymphocytes; MCAO, middle cerebral artery occlusion; mRNA messenger ribonucleic acid; MLN, mesenteric lymph node; PP, Peyer’s patches; SIgA, secretory IgA; TCR, T cell receptor; TNF, tumor necrosis factor; CB1R ko, cannabinoid 1 receptor knock out; Mix, mixing newborns from distinct litters; ETEC, enterotoxigenic E. coli.*

## THE NERVOUS SYSTEM AND THE INTESTINAL IMMUNE RESPONSE

The mutual influence of the nervous system and the intestinal immune response has been widely studied using experimental models of stress. ILs and microbiota from the gut can modulate the nervous system. On the other hand, the nervous system can modulate intestinal immunity by several pathways (de Jonge, 2013).

**FIGURE 1:**
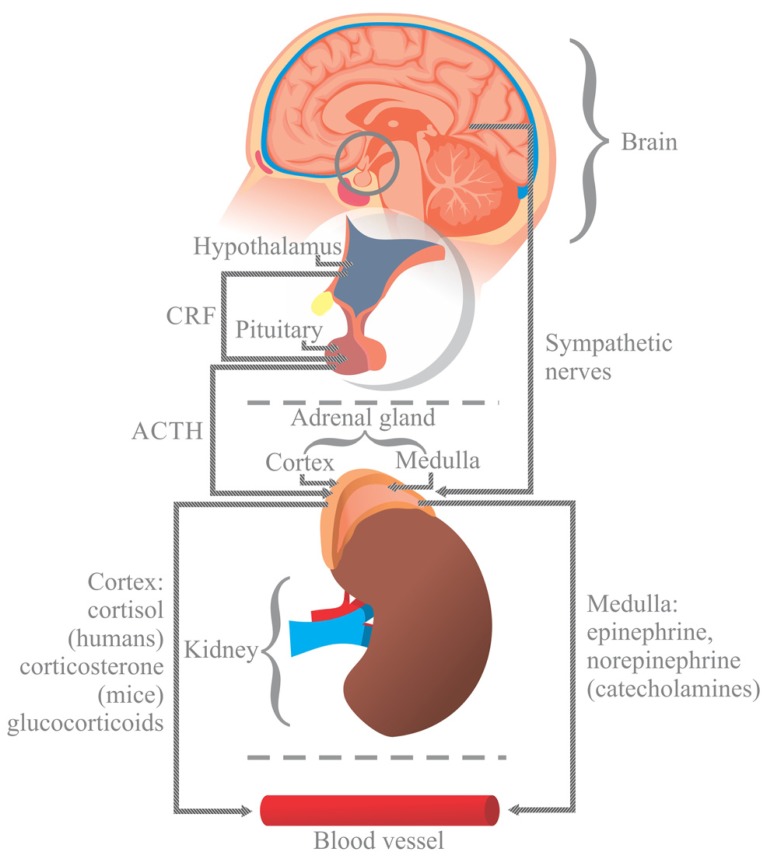
**In response to stress, the hypothalamus (H) releases the corticotrophin releasing factor (CRF) into the anterior pituitary (P), causing the release of adrenocorticotropic hormone (ACTH) into the blood flow.** ACTH stimulates the generation of glucocorticoids (cortisol in humans and corticosterone in mice) in the cortex of the adrenal gland (A), which are then released into the blood. Stress also activates the autonomic sympathetic nerves in the medulla of the adrenal gland to elicit the production of catecholamines, norepinephrine and epinephrine, which are then released into the blood. Glucocorticoids and catecholamines influence the generation of interleukins, which are involved in the viability and proliferation of immunocompetent gut cells via receptors.

For instance, the nervous system regulates immune function through the hypothalamic pituitary adrenal axis (HPA). In response to stress, the hypothalamus releases the corticotrophin releasing factor (CRF) into the anterior pituitary, causing the release of adrenocorticotropic hormone (ACTH) into the blood flow. ACTH stimulates the generation of glucocorticoids (cortisol in humans and corticosterone in mice) in the cortex of the adrenal medulla (McClennen et al., 1998; Jezova et al., 1999), which are then released into the blood. Additionally, the nervous system modulates intestinal immunity through the sympathetic autonomic nervous system by triggering the release of catecholamines (adrenaline and noradrenaline) from the adrenal gland medulla (Kvetnanský, 1995; Jezova et al., 1999; **Figure 1**).

**FIGURE 2:**
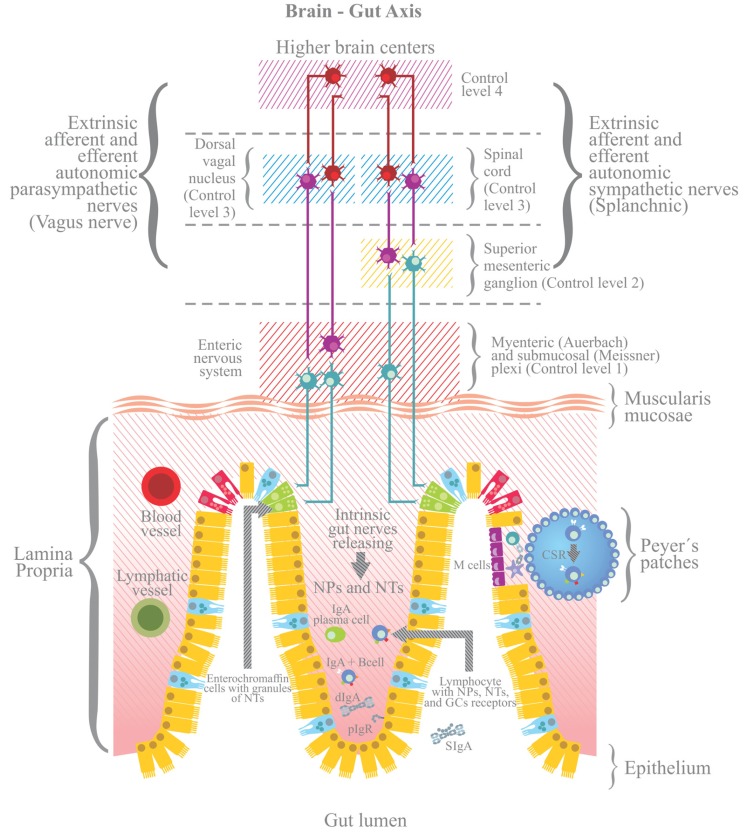
**Stress also triggers the activation of the enteric nervous system, including afferent and efferent intrinsic intestinal nerves (afferent nerves send signals from periphery to the brain; efferent nerves from the brain to the periphery) and extrinsic innervations, whether sympathetic (splanchnic) or parasympathetic (Vagus nerve).** The enteric nervous system is connected to the CNS via sympathetic and parasympathetic pathways, forming the brain-gut axis (BGA). Four levels for the control of BGA are shown (Wood et al., 1999). The stress response of the BGA influences the generation of dIgA and/or the pIgR mediated trancytosis. NTs, neurotransmitters; NPs, neuropeptides; GCs, glucocorticoids.

Stress also triggers the response of a plethora of peptide hormones and neurotransmitters by intrinsic innervation of the ENS. The ENS is connected bidirectionally to the CNS through sympathetic and parasympathetic nerve pathways forming the brain-gut axis (BGA; de Jonge, 2013). The autonomic ENS comprises sympathetic (noradrenergic) and parasympathetic (cholinergic) fibers that interact directly with the CNS through parasympathetic (vagal) and sympathetic splanchnic fibers. Within the ENS the intrinsic nerve fibers are organized in myenteric (Auerbach’s plexus), submucosal (Meissner’s plexus) and mucosal plexus. The latter contain nerve endings that can make contact with antigen presenting cells (APCs) to control gut immune responses (de Jonge, 2013). Regulation of the BGA is accomplished by the integration of four control levels. Control level one is accomplished by the ENS endowed with local innervations that functionally are independent of the extrinsic nervous connections. Level two entails the prevertebral sympathetic ganglia where peripheral reflex pathways are influenced by preganglionic sympathetic fibers from the spinal cord. Levels three and four are within the CNS. In the level three sympathetic and parasympathetic fibers outflow to the gut is determined in part by reflex with sensory fibers that travel with autonomic nerves. The level four includes higher nerve centers that supply descending signals that are integrated with incoming sensory signals at the level three (**Figure 2**).

At the intestinal level the bilateral communication between the nervous system and intestinal immune response occurs through sympathetic innervations, which influence (i) the differential distribution of immunocytes in different regions of the small intestine (Ke et al., 2011), (ii) the migration of lymphocytes towards Peyer’s patches and mesenteric lymphoid nodules (MLN; Gonzalez-Ariki and Husband, 1998), and (iii) the ontogeny of IgA+ B cells populating the intestinal lamina propria (Gonzalez-Ariki and Husband, 2000).

Mouse gut is innervated by fibers expressing adrenergic receptors (Hirafuji et al., 2001; Nasser et al., 2006). Upon interacting with an agonist, these receptors enable enterochromaffin cells to release neuropeptides that affect IgA levels. The secretion of IgA is also influenced by the interaction of peptidic innervations inside Peyer’s patches with immunocytes, and of nerve fibers from the gut basement membrane with IgA+ cells (Hisajima et al., 2005; Vulchanova et al., 2007). Moreover, the release of norepinephrine (Schmidt et al., 2007), acetylcholine (Wilson et al., 1982; Schmidt et al., 2007) and neuropeptides (Schmidt et al., 1999) by gut nerve fibers modulates the secretion of intestinal IgA and the expression of pIgR (Cox et al., 2007). Additionally, gut nerve fibers release the vasoactive intestinal peptide, neuropeptide Y (Mongardi Fantaguzzi et al., 2009) and somatostatin (Schmidt et al., 1999), which all help modulate the intestinal production of SIgA (Shibata et al., 2008).

## THE GENERATION OF INTESTINAL IgA

From an immunological point of view, intestinal SIgA is produced by a multistage process modulated by ILs. This process involves the activation and class switch recombination of IgM+ to IgA+ B cells, the latter of which are committed to IgA synthesis either by a T-cell dependent or T-cell independent pathway (Cerutti, 2008).

**FIGURE 3:**
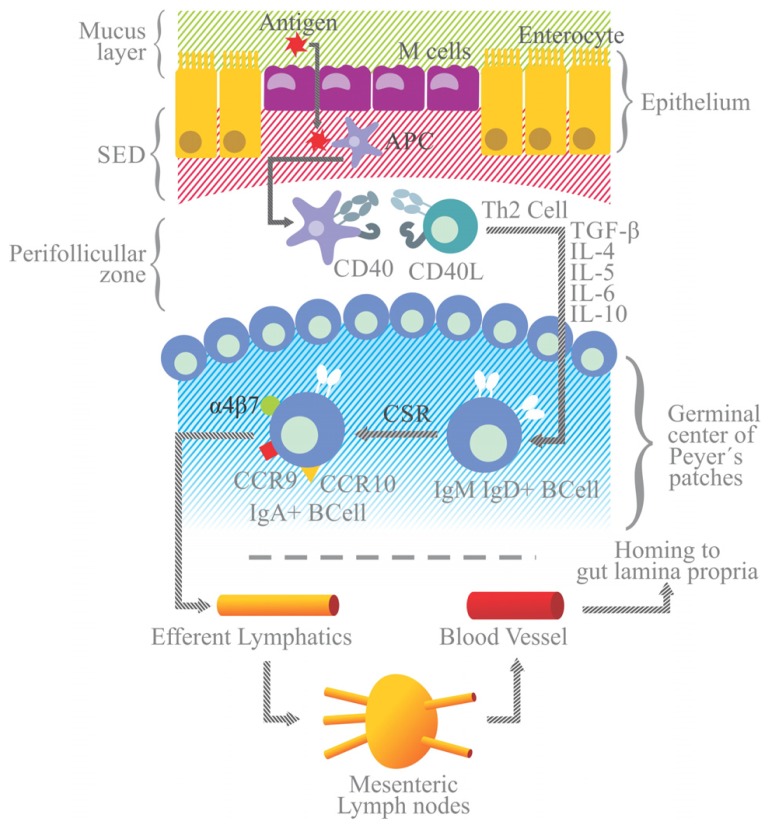
**Simplified model of IgA generation.** Antigen presenting cells and dendritic cells are located in the subepithelial dome (SED) beneath M cells. After sampling, luminal antigen is processed and expressed on the surface as an antigen-derived peptide associated with the MHC-II molecule. Dendritic cells with the MCH II peptide and the CD40 antigen on the surface migrate to the perifollicular zone of Peyer’s patches to interact with Th2 lymphocytes endowed with TCRs and CD40 ligand (CD40L) on their surface. Immunological synapsis between APC and Th2 cells via the binding of the MHC-II peptide to TCR and of CD40 to CD40L leads to the activation of Th2 and the release of TGF-β. Along with signals elicited by the CD40/CD40L binding, TGF-β is a Th2-derived IL essential for the class switch recombination of IgM+ B cells to generate IgA+ B cells, occurring in the germinal center of Peyer’s patches (highlighted in blue). Other Th2 ILs (e.g., IL-4, -5, -6, and -10) favor the differentiation and maturation of IgA+ B cells into IgA+ plasma cells. IgA+ B lymphocytes acquire the expression of gut-homing receptors, such as α4β7, CCR9 and CCR10, and migrate toward efferent lymphatic vessels and mesenteric lymphoid nodules. From there they can reach the blood stream and migrate to the lamina propria (the gut effector site). Gut epithelial cells express the MadCAM1 ligand for α4β7, and release CCL25 and CCL28, chemokines that are ligands for the CCR9 and CCR10 receptors, respectively.

The T-dependent pathway is induced in follicular areas of Peyer’s patches after the interaction between the APCs and helper Th2 lymphocytes (**Figure 3**). On their surface, APCs (like dendritic cells) express the CD40 antigen and a peptide-derived antigen associated with the major histocompatibility class II molecule (MHC-II). The CD40 antigen interacts with CD40L on Th2 cells, and the peptide-MHC-II complex with the Th2 cell receptor (TCR). In either case, immunological synapses lead to the release of the transforming growth factor (TGF)-β1 by Th2 cells, which is an essential step for the activation and class switch recombination of IgM+ B cells to IgA+ B lymphocytes.

Other Th2-derived ILs, including IL-4,-5,-6, and -10, promote the proliferation of IgA+ B cells and their differentiation into IgA secreting plasma cells. In the presence of retinoic acid, IgA+ B cells express gut-homing receptors, such as α4β7 integrin, CCR9 and CCR10, and cause these cells to migrate from Peyer’s patches to the MLN via the circulation of efferent lymphatic vessels. From the MLN these cells go to the thoracic duct, enter the bloodstream, and finally home to the lamina propria, the effector site of the gut immune system.

Epithelial cells that line the lamina propria express mucosal address in cell adhesion molecule 1 (MadCAM1) and the chemokines CCL25 and CCL28, which are the ligands for α4β7 integrin, CCR9 and CCR10, respectively, on B cells. In the lamina propria IgA+ B cells mature to plasma cells capable of releasing dimers or polymers of IgA joined to J-chain (Brandtzaeg, 2009; **Figure 3**).

The T-independent pathway for the production of intestinal IgA occurs in extra-follicular structures, including isolated lymphoid follicles and lamina propria. The class switch recombination of IgM+ B cells to IgA+ B lymphocytes takes place through two pathways, and both involve a T-independent antigen. IgM+ B lymphocytes express B cell receptors (BCRs) and Toll-like receptors (TLRs). Polysaccharides interact with BCRs and bacterial lipopolysaccharide (LPS) and/or nucleic acids with TLRs (Cerutti, 2008). In the lamina propria IgA+ B cells further differentiate into IgA+ plasma cells.

## THE TRANSPORT OF INTESTINAL IgA

The expression of pIgR is necessary for the transport (transcytosis) of dIgA or pIgA across the epithelial layer. pIgR is a 120 kDa transmembrane protein consisting of five extracellular immunoglobulin (Ig) homology domains, a transmembrane region and a cytoplasmic domain. The amino (NH_2_) terminal of this protein chain is oriented to the extracellular space, while the carboxyl (COOH) terminal has an intracellular orientation and contains signals for intracellular sorting and endocytosis (Asano and Komiyama, 2011; Johansen and Kaetzel, 2011).

pIgR is expressed on the basolateral surface of epithelial cells. Its expression can be constitutive or regulated at a transcriptional level by IL-4 and pro-inflammatory cytokines, the latter including tumor necrosis factor α (TNF-α) and interferon γ (IFN-γ; Johansen and Kaetzel, 2011). IgA transcytosis begins when pIgR uptakes dIgA or pIgA released in the lamina propria by plasma cells (Cerutti, 2008). The dIgA-pIgR or pIgA-pIgR complex is transported by vesicles across the epithelial cell, and upon reaching the apical side pIgR is cleaved to render SC bound to dIgA/pIgA. The resulting SIgA is released into the intestinal lumen (Asano and Komiyama, 2011).

**FIGURE 4:**
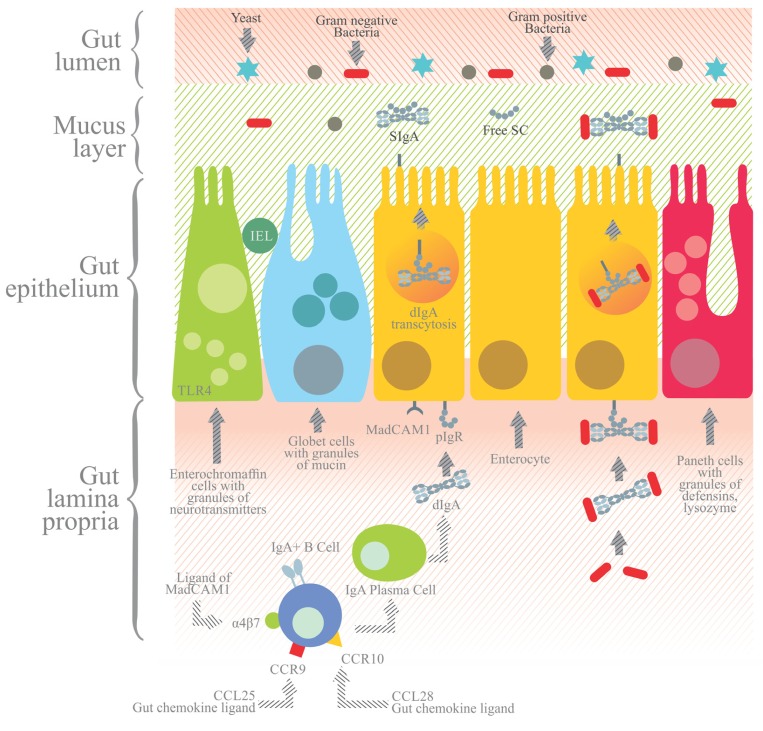
**In the gut lamina propria, IgA+** B cells further differentiate into IgA**+** plasma cells that secrete dIgA or pIgA antibodies joined by the J chain. dIgA or pIgA are captured by the polymeric immunoglobulin receptor (pIgR), a transmembrane receptor with five extracellular domains and an intracellular tail expressed at the basolateral side of the enterocytes. The dIgA-pIgR complex is internalized and transported by transcytosis to the apical side, and the extracellular portion of pIgR with five domains is proteolytically cleaved from the transmembrane region. The former is then released to the gut lumen as secretory component (SC) bound to dIgA to yield secretory IgA (SIgA). SIgA and SC secreted into the mucus layer prevent the direct adhesion to the epithelium of pathogenic agents, which are eventually cleared from the lumen. Apart from enterocytes, other cell components of the gut epithelium include enterochromaffin cells with granules of neurotransmitters, Paneth cells with granules containing defensins and lysozyme, and goblet cells with mucin granules and IEL.

The cleavage of pIgR to yield SC occurs at the linker that connects domain 5 to the transmembrane region (**Figure 4**). Biochemically and morphologically, trancytosis involves: (i) the endocytosis of dIgA from clathrin coated pits and its delivery to basolateral endosomes, (ii) microtubule dependent translocation to apical recycling endosomes, and (iii) delivery of the plasma membrane to apical endosomes (Johansen and Kaetzel, 2011).

## THE ROLE OF SIgA AND pIgR

Both SIgA and pIgR have an essential role in immune exclusion, which protects against infections caused by enteropathogens. This role has been explored in the murine model of *Salmonella* *typhimurium* infection (Michetti et al., 1992; Drago-Serrano et al., 2010).

SIgA helps to limit the adhesion of luminal antigens to the epithelium. These antigens, if not excluded in gut secretions, are able to elicit the release of cell derived inflammatory cytokines, which can enhance permeability and disrupt the functional integrity of the gut. As a result of increased gut permeability, penetration of luminal antigens into the systemic compartment may cause a strong and even life-threatening systemic inflammatory response (Brandtzaeg, 2009; Corthésy, 2007).

pIgA and the different forms of IgA also have an anti-inflammatory role. For instance, pIgA and dIgA protect host tissue by neutralizing pro-inflammatory antigens inside and below the gut epithelium layer. On the other hand, dIgA and pIgA are unable to elicit the production of pro-inflammatory cytokines on cells by binding with receptors specific for the Fc α-domain (Corthésy, 2007). Furthermore, SIgA and pIgR, along with the intestinal microflora, contribute to gut homeostasis by maintaining the intestinal inflammatory response within the normal physiological limit (Uren et al., 2003; Sait et al., 2007; Bruno et al., 2010). Since the gut microbiota and SIgA are bilaterally modulated, an alteration in one may affect intestinal homeostasis and lead to intestinal inflammation (Suzuki et al., 2004; Bruno et al., 2010).

Hence, the generation of IgA+ B cells in Peyer’s patches, the homing of IgA+ B cells to the gut lamina propria, and the transcytosis of dIgA/pIgA via pIgR are all potential targets of stress-related effects that can alter SIgA levels.

## THE RESTRAINT MODEL AND THE INFLUENCE OF STRESS IN THE INTESTINE

Assays based on the restraint model have provided important insights into the influence of stress on the humoral and cellular components involved in the intestinal immune response via neuroendocrine pathways. In the restraint procedure, a rodent is placed (without forced squeezing) inside a cylindrical plastic tube. This represents mainly psychological stress, as the perception of confinement mimics a collapsed tunnel for these burrow-dwelling animals (Dhabhar and Viswanathan, 2005). Another restraint procedure, known as immobilization, involves adhering outstretched rodent limbs on a board with tape. Compared to restraint in a plastic tube, this model has elicited a much more robust stress response through the generation of neuroendocrine mediators (e.g., glucocorticoids and catecholamines; Glavin et al., 1994).

The restraint stress model has provided evidence of intricate neurological pathways underlying the regulation of SIgA. Such pathways involve neurotransmitters and endocrine hormones released from the blood flow or produced locally (e.g., glucocorticoids released by intestinal epithelial cells), and their interaction with the receptors of target cells (Cima et al., 2004).

It is now known that modulation of SIgA production is influenced by the duration (acute or chronic) and intensity of stress. Generally at a systemic level, acute stress (represented by a single session lasting a few minutes to a few hours) tends to upregulate the number of immune cells. Contrarily, the multiple sessions over a period of several days, weeks or months that represent chronic stress (Dhabhar, 2009) tend to downregulate the systemic immune response. Experimental assays with skin delay type hypersensitivity response in rats corroborate this same general pattern of acute and chronic stress (Dhabhar and McEwen, 1997).

Intensity of stress is another factor that can influence the result on the immune response. It is measured by the increase in levels of adrenal hormones, neurotransmitters and physiological parameters (e.g., heart rate and blood pressure), as well as the period of time that these changes persist (during and after the stress-inducing event; Dhabhar, 2009). This assays on mice have shown that (i) the acute stress response elicited by intense running promotes the accumulation of T lymphocytes in Peyer’s patches via adrenergic mechanisms, evidenced by the fact that this exercise-dependent increase was blocked by α- (phentolamine) or β- (nadolol) adrenoceptor antagonists (Krüger et al., 2008), and (ii) repeated sessions of chronic restraint stress have a negative influence on intestinal levels of SIgA, which may be due to the capability of corticosteroids to decrease the trancytosis of SIgA via pIgR (Jarillo-Luna et al., 2007). Another study on mice reported that corticosteroids decrease pIgA levels in mucosal secretions and increase such levels in serum. This effect is due in part to the greater production in serum of SC, which is derived from hepatic pIgR. The binding of SC to pIgA retards the clearance of the latter from the blood by the liver (Wira et al., 1990).

In addition to reducing intestinal SIgA levels, repeated stress has a negative influence on the number of lymphoid cells in Peyer’s patches and the intestinal intraepithelial compartment. These suppressive effects of restraint stress were mimicked by the administration of pharmacological dose of catecholamines and glucocorticoids, results suggested the activation of two pathways–- the sympathetic autonomic nervous system and the HPA axis (Jarillo-Luna et al., 2007, 2008; Martínez-Carrillo et al., 2011). In another study, the endogenous production of glucocorticoids, triggered by a continuous 12-h period of restraint stress, decreased the number of T and B cells in Peyer’s patches, which is in line with a reduction in the levels of intestinal SIgA and lymphocytes (Sudo et al., 2001).

Other experimental stress protocols have been reported to have a negative influence on the gut immune response (**Table 1**). The stress produced in rats by exposure to heat negatively affected some intestinal parameters, including the levels of CD3+ and CD4+ T lymphocytes, the expression of TLR-2 and TLR-4, as well as the transcriptional mRNA expression of IFN-γ and IL-2, -4, and -10 (Liu et al., 2012). Assays on rats with an electric shock protocol showed that stress suppressed the production of IFN-γ through T cells with TCRαβ in the intraepithelial compartment, while at the same time elevating the level of endogenous glucocorticoids (Zhang et al., 2005). Studies on rodents under psychological stress have also reported a decrease in levels of intestinal SIgA, caused by: (i) an expectation of electric foot shock (Yamamoto et al., 2009), (ii) a continuous back and forth transference from housing cages to metabolic cages (Eriksson et al., 2004), and (iii) immobilization, in some cases combined with exposure to loud noise (Ponferrada et al., 2007; Caso et al., 2009; Zoppi et al., 2012).

## THE IMMUNOMODULATORY MECHANISMS OF STRESS (**Table 2**)

**Table 2:** Mechanisms of immune modulation by stress.

Effect	Mechanism	Reference
↓ SIgA levels by acute immobilization stress	↓ SIgA attenuated by peroxisome proliferator-activated receptor- γ (PPAR)-γ activation	Ponferrada et al. (2007)
↓ SIgA levels by immobili-zation and acoustic stress	↓ SIgA attenuated by cannabinoid 1 receptor (CB1R) activation	Zoppi et al. (2012)
↓ activation/migration of T cells induced by restraint stress	Alterations of cytoskeletal actin and plasma membrane factors by stress hormones	Flint et al. (2011)
↓ number of lymphocytes in spleen by restraint stress	Apoptosis through p53 and PI3K/NF-κB pathways	Zhang et al. (2008a)
↓ number of T lymphocytes in Peyer’s patches by exercise associated stress	Fas/FasL apoptosis pathway	Krüger et al. (2009)
↓ number of lymphocytes in spleen by chronic restraint stress	μ-receptor mediated apoptosis, dependent on endogenous opioids and independent of glucocorticoids from activation of HPA axis	Wang et al. (2002)
↓ number of splenocytes by chronic restraint stress	CD95 (Fas/APO-1) mediated apoptosis, dependent on endogenous opioids but independent of the activation of HPA axis	Yin et al. (2000)
↑ Immunosuppression by chronic restraint stress	Apoptosis via TLR4/PI3K signaling	Zhang et al. (2008b)
↑ Immunosupression by restraint stress	↓ MHC-II expression in peritoneal macrophages along with ↑corticosterone levels	Zwilling et al. (1990)
↑ Immunosuppression by restraint stress	↓ MHC-II expression influenced by corticosterone and some hormones not associated with the activation of HPA axis	Zwilling et al. (1993)
↑ T cell proliferation or apoptosis by restraint stress	Activation of *GADD45g *and *pura* genes, responsible for apoptosis and proliferation, respectively	Flint et al. (2005)
↑ SIgA levels and ETEC proliferation following stress by weaning and short term exposure to cold	Catecholamines enable iron acquisition that promotes bacterial proliferation	Jones et al. (2001), Lyte et al. (2011)

*↓ down modulation; ↑up modulation; MHC-II, major histocompatibility complex class II; PI3K/NF-κB, phosphoinositide 3 kinase/nuclear factor κB; TLR4, Toll-like receptor 4; HPA, hypothalamus-pituitary-adrenal axis; GADD45g, growth arrest and DNA-damage inducible gamma; pura, purine rich element binding protein A; ETEC, Enterotoxigenic Escherichia coli; SIgA, secretory immunoglobuline A.*

### CELL MIGRATION

The aforementioned stress-related changes in the levels of IgA, IgA secreting cells and IgA producing ILs may be related to cell migration, a phenomenon that has been studied mostly in the systemic response (Bauer et al., 2001). Acute restraint stress decreases the number of peripheral helper T lymphocytes, upregulates the expression of adhesion molecules (CD11a and CD11b) on T cells, and increases the levels of circulating NK cells and glucocorticoids. These changes were not found in mice previously exposed to chronic intermittent restraint stress, suggesting an adaptation response to prolonged stress.

One presumable mechanism entails greater restraint-induced levels of glucocorticoids and/or catecholamines, which influence lymphocyte trafficking through the expression of adhesion molecules on endothelial cells (Dietrich, 2004; Krüger and Mooren, 2007; Krüger et al., 2008). This effect was found in mice under stress caused by exercise (Krüger et al., 2008). It seems that stress hormones influence lymphocyte migration and function through specific alterations in the actin cytoskeleton, an effect also found in mice under restraint stress (Flint et al., 2011).

### CELL VIABILITY AND T CELL ACTIVATION

Another downmodulatory mechanism related to restraint stress is a decrease in cell viability and/or T cell activation. For instance, at a systemic level the restraint stress protocol elicits a reduction in splenic lymphocytes by apoptosis through the activation of p53 and PI3K/NFκB pathways (Zhang et al., 2008a). p53 is a pro-apoptotic factor which upmodulates the expression of Fas. Phosphoinositide 3 kinases (PI3K) are signal transduction enzymes involved in regulating cell proliferation, and the nuclear factor κB (NFκB) modulates the expression of genes involved in the innate and adaptive immune responses, as well as in cell survival and death (Zhang et al., 2008a).

At the intestinal level, the decreased number of viable lymphocytes in Peyer’s patches induced by restraint stress may also lead to apoptosis (Sudo et al., 2001), which seems to be dependent on the Fas/Fas ligand activation signal, as evidenced by T cells in Peyer’s patches of mice under stress by intense exercise (Krüger et al., 2009). One report suggested that glucocorticoids are the main apoptotic inducers involved in the decreased number of intestinal intraepithelial lymphocytes (Brunner et al., 2001), which is in agreement with other studies. The molecular mechanisms of glucocorticoid-induced apoptosis are highly dependent on the binding of this ligand with its receptor (Schmidt et al., 2004), which is a cytosolic ligand-dependent transcription factor. After binding to the ligand, the glucocorticoid receptor dissociates from the protein complex, dimerizes and translocates into the nucleus, where it then binds to regulate the transcription of apoptotic genes (Schmidt et al., 2004).

Another presumable mechanism of lymphocyte apoptosis induced by restraint stress relies on signals triggered by the interaction of endogenous opioids with μ-opioid (Wang et al., 2002) and CD95 receptors (Yin et al., 2000). Cell death caused by the binding of endogenous opioids with CD95 (also known as Fas or apo1) or μ-opioid seems to be independent of the HPA axis (Yin et al., 2000). The binding of CD95 with specific agonists induces the activation of a cascade of caspases, and ultimately nucleases, that results in apoptotic cell death (Yin et al., 2000). Endogenous opioid peptides (endorphins, enkephalins and endomorphins) play a critical modulatory role in emotional stress-induced changes in the immune system (Bodnar, 2012).

An additional mechanism by which restraint stress downmodulates lymphocytes is through the activation of TLR-4, which in turn inhibits the activation of the PI3K (Zhang et al., 2008b). While inhibition of the PI3K signaling pathway induces lymphocyte apoptosis (Yin et al., 2006), its activation inhibits the same (Wu et al., 2000). TLR-4 can also mediate signaling that leads to cell death through the interaction of the death domain of myeloid differentiation factor 88 (MyD88) with the Fas associated death domain (FADD; Haase et al., 2003).

### THE INHIBITION OF MHC-II

Expression of the MHC-II glycoprotein by APC is essential for the initiation of the immune response by CD4+ T cells (Blum et al., 2013). A stress-induced decrease in MHC-II expression is carried out by elevated levels of corticosterone (Zwilling et al., 1990) and other hormones not associated with the HPA axis (Zwilling et al., 1993). It seems that a higher level of corticosterone triggered by restraint stress diminishes the number of IFN-γ receptors on macrophages. Since the binding of lFN-γ to its receptor triggers the signaling necessary for MHC-II expression (Zwilling et al., 1992), a reduction in the expression of this receptor decreases the expression of MHC-II.

### ENDOGENOUS RECEPTORS CAN ATTENUATE THE STRESS-INDUCED DOWN REGULATION OF SIgA

Assays conducted on mice under a protocol of immobilization, in some cases with exposure to loud noise, evidenced that the immunosuppressive influence of stress on SIgA can be attenuated by the activation of the cannabinoid 1 receptor (CB1R; Zoppi et al., 2012) and the peroxisome proliferator-activated receptor (PPAR)-γ (Ponferrada et al., 2007). Both the CB1R (Hill and McEwen, 2010) and PPAR-γ nuclear receptors (Dubuquoy et al., 2006) have an essential role in the modulation of colon inflammation by stress.

Cannabinoid 1 receptor is one of the most prominent receptors for cannabinoids distributed in the CNS and peripherally in immune cells. It is a G-protein coupled receptor whose endogenous ligands are arachidonate derived lipophilic molecules, N-arachidonylethanolamine anandamide and 2- arachidonylglycerol, which affect emotional behavior (Hill and McEwen, 2010).

Peroxisome proliferator-activated receptor-γ is a nuclear receptor expressed in the colon that forms a heterodimer with the retinoid X receptor (RXR). It is activated by natural endogenous ligands, polyunsaturated fatty acids (PUFAs) and eicosanoids, allowing for its heterodimerization with RXR and its binding to the nuclear peroxisome proliferator response element (PPRE). PPAR-γ and RXR play a central role in the regulation of inflammatory signaling pathways by acting on kinases and transcription factors, such as NFkB, and by inhibiting mucosal production of inflammatory cytokines (Dubuquoy et al., 2006). When PPAR-γ expression in intestinal epithelial cells is induced by LPS-activated TLR-4, it leads to the regulation of NFkB and MAPK pathways and modulation of the inflammatory response. Up regulation of TLR-4 expression together with impaired expression of PPAR-γ in epithelial cells may lead to superficial colonic inflammation in patients with ulcerative colitis.

### THE UPMODULATORY EFFECTS OF STRESS

Although stress has been regarded as immunosuppressive, it can enhance the levels of IgA (Yamamoto et al., 2009) and CD3+/CD8+ T lymphocytes in the MLN of rats under stress by electric foot shock or exposure to heat (Liu et al., 2012). The capacity of restraint stress to activate the gene expression of purine rich element binding protein A (*pura*) has been reported to be responsible for priming T cells to undergo proliferation (Flint et al., 2005). Thus, the upmodulatory effects of restraint stress hormones on SIgA levels and on mRNA expression of pIgR should not be surprising (Reyna-Garfias et al., 2010). Indeed, at the molecular level it has been reported that glucocorticoids upmodulate the transcriptional mRNA expression of pIgR via a glucocorticoid DNA response element located in the 5’upstream region of the pIgR gene in rat duodenum (Li et al., 1999).

Experimental studies have evidenced that stress can trigger SIgA secretion in response to an enhanced bacterial proliferation, as reported in feces from piglets infected with ETEC under protocols involving weaning and short-term exposure to cold (Jones et al., 2001). In this case, the presumable influence of stress in promoting bacterial proliferation may be related to catecholamines, which can make iron available to bacteria by removing it from host proteins like transferrin and lactoferrin (Lyte et al., 2011).

Although the context of the development of the immune response in the intestine and systemic compartments is different, the modulatory influence of the stress response may share some mechanisms in both cases.

## CONCLUSION AND PERSPECTIVES

Gut homeostasis results from neuroimmune modulation by anti- and pro-inflammatory ILs, neurotransmitters and endocrine hormones, all of which influence the generation of intestinal SIgA. This immunoglobin in turn affects intestinal inflammation and permeability, which are essential factors in the functional integrity of the gut under stress conditions. Experimental studies with the restraint/immobilization rodent model have resulted in an up or down modulation of SIgA levels depending on the intensity and time of exposure to stress. In the case of down regulation, there is an increased susceptibility to infection and intestinal inflammation. Pharmacological modulation of the cannabinoid system and the PPAR-γ may be therapeutically useful for intestinal dysfunctions resulting from a stress-induced decrease in SIgA levels. Future studies should explore the adaptation of experimental models for the evaluation of therapeutic and preventive strategies to control intestinal inflammation and/or infection in patients with high vulnerability to stress.

## Conflict of Interest Statement

The authors declare that the research was conducted in the absence of any commercial or financial relationships that could be construed as a potential conflict of interest.
